# Effects of vitamin D supplementation on androgens in men with low testosterone levels: a randomized controlled trial

**DOI:** 10.1007/s00394-018-1858-z

**Published:** 2018-11-20

**Authors:** Elisabeth Lerchbaum, Christian Trummer, Verena Theiler-Schwetz, Martina Kollmann, Monika Wölfler, Annemieke C. Heijboer, Stefan Pilz, Barbara Obermayer-Pietsch

**Affiliations:** 1 Division of Endocrinology and Diabetology, Department of Internal Medicine, Medical University of Graz, Auenbruggerplatz 15, 8036 Graz, Austria; 2 Division of Gynecological Endocrinology and Reproductive Medicine, Department of Obstetrics and Gynecology, Medical University of Graz, Auenbruggerplatz 14, 8036 Graz, Austria; 3 Endocrine Laboratory, Department of Clinical Chemistry, VU University Medical Center, De Boelelaan 1117, 1081 HV Amsterdam, The Netherlands; 4 Laboratory of Endocrinology, Academic Medical Center, Meibergdreef 9, 1105 AZ Amsterdam, The Netherlands

**Keywords:** Vitamin D, Testosterone, Randomized controlled trial, SHBG, Androgens

## Abstract

**Purpose:**

It has been hypothesized that vitamin D is associated with androgen levels in men. We, therefore, aimed to evaluate whether vitamin D supplementation increases serum total testosterone (TT) levels in men with low TT levels at baseline.

**Methods:**

The Graz Vitamin D&TT-RCT is a single-center, double-blind, randomized placebo-controlled trial conducted between March 2013 and November 2017 at the endocrine outpatient clinic at the Medical University of Graz, Austria. One-hundred healthy men with serum TT levels < 10.4 nmol/l and 25-hydroxyvitamin D [25(OH)D] levels < 75 nmol/l participated in the trial. Subjects were randomized to receive 20,000 IU of vitamin D3/week (*n* = 50) or placebo (*n* = 50) for 12 weeks. Primary outcome was TT measured using mass spectrometry. Secondary outcomes were free testosterone, free androgen index, sex hormone-binding globulin, estradiol, follicle-stimulating hormone, luteinizing hormone, metabolic characteristics, and body composition.

**Results:**

Ninety-four men [mean age and 25(OH)D: 47 (± 12) years and 56.3 (± 18.3) nmol/l, respectively] completed the study. We found no significant treatment effect on serum TT or on the remaining secondary outcome variables.

**Conclusion:**

Vitamin D treatment had no effect on serum TT levels in middle-aged healthy men with low TT levels.

## Introduction

Vitamin D is well known for its role in maintaining calcium homeostasis and promoting bone mineralization [1]. Considering the high prevalence of an insufficient vitamin D status in many populations as well as the potential link between low vitamin D status and adverse health outcomes [2], vitamin D deficiency is classified as an important public health problem [1]. Beyond the association between vitamin D deficiency and musculoskeletal diseases, evidence is accumulating that vitamin D deficiency is also a risk marker for insulin resistance [3], cardiovascular disease [4], infectious and autoimmune diseases [2], cancer [5] as well as increased mortality [1]. Likewise, low testosterone levels in men are related to adverse events including increased cardiovascular and all-cause mortality [6–9]. As men with combined androgen and vitamin D deficiencies are at high risk for mortality, a parallel deficiency of both hormones has been suggested to be a marker of poor overall health [10]. Therefore, a causal relationship between vitamin D and testosterone [11] is of high clinical interest. In particular, a potential increase of testosterone levels after vitamin D treatment might be important.

Testosterone is produced in the Leydig cells following pituitary pulsatile LH secretion. Its production is also modulated by paracrine and autocrine signals supplied by growth factors and cytokines secreted within the testis [12, 13]. The vitamin D receptor (VDR) is almost ubiquitously expressed in human cells, which underlines the clinical significance of the vitamin D endocrine system [1, 2, 14]. VDR- and vitamin D-metabolizing enzymes are concomitantly expressed in the entire reproductive male tract, including Leydig cells [15]. Further, vitamin D significantly increased testosterone production in a human primary testicular cell culture model [11]. Therefore, vitamin D might be involved in the production of male reproductive hormones.

Observational studies have, by the majority, shown that vitamin D deficiency is associated with low testosterone concentrations [16]. In contrast, data from randomized controlled trials (RCTs) on vitamin D supplementation and testosterone status have consistently shown no statistically significant effect of vitamin D vs. placebo regarding testosterone levels [16]. Likewise, we failed to find a significant effect of vitamin D treatment on androgen levels in healthy men with normal total testosterone (TT) levels participating in the Graz Vitamin D&TT-RCT, a RCT recruiting 100 men with normal serum TT concentrations and 100 men with low serum TT concentrations [17].

In this manuscript, we present results from men with low serum TT levels who participated in the Graz Vitamin D&TT-RCT but required longer recruitment time compared to men with normal serum TT concentrations thus resulting in two separate publications. To our knowledge, this is the first RCT specifically designed to analyze vitamin D effects on androgen levels in men with low baseline serum TT levels.

## Methods

### Study design

We present the results of the second arm (involving men with low serum TT levels) of the Graz Vitamin D&TT-RCT, a single-center, double-blind, placebo-controlled, parallel-group study performed at the Medical University of Graz, Austria. The trial was designed to investigate the effect of vitamin D supplementation (12 weeks) on serum TT levels in men. The methods and study design have been published in detail previously [17]. The design, conduction and publication of this study adhere to the recommendations of the CONSORT Statement (http://www.consort-statement.org/). The trial was registered at http://www.clinicaltrialsregister.eu (EudraCT number, 2011-003575-11) and at clinicaltrials.gov (ClinicalTrials.gov Identifier NCT01748370). The study protocol was approved by the ethics committee of the Medical University of Graz (EK 23-513 ex 10/11) and written informed consent was obtained from each participant before entering the study.

The Graz Vitamin D&TT-RCT examines vitamin D effects in 100 men with normal serum TT levels (results have been published previously [17]) as well in 100 men with low serum TT levels (data presented in this manuscript).

### Subjects

Eligible study participants were men aged ≥ 18 and < 70 years with 25-hydroxyvitamin D [25(OH)D] levels < 75 nmol/l and serum TT levels < 10.4 nmol/l. Exclusion criteria were hypercalcemia (defined as a serum calcium > 2.65 mmol/l), oral or transdermal testosterone supplementation in the last 2 months before entering the study, intramuscular testosterone supplementation 6 months before entering the study, regular intake of vitamin D supplements before study entry, chronic diseases (such as diabetes mellitus), thyroid disease, endocrine disturbances in need of treatment (such as pituitary disorders), history of hypogonadisms or known diseases associated with hypogonadism (except obesity) or diseases known to interfere with vitamin D intake or sensitive to vitamin D intake (including inflammatory diseases with granuloma such as sarcoidosis, tuberculosis, Wegener’s granulomatosis; including other forms of vasculitis and inflammatory bowel diseases), intake of medication influencing metabolic or endocrine parameters (insulin sensitizers, insulin, or glucocorticoids) in the last 3 months before study entry; PSA > 4 ng/ml (or > 3 ng/ml in men at high risk for prostate cancer), palpable prostate nodule or induration, hematocrit > 50%, untreated severe obstructive sleep apnea, severe lower urinary tract symptoms, uncontrolled or poorly controlled heart failure, a history of prostate cancer, breast cancer, orchidectomy, and chromosomal disorders (e.g. Klinefelter Syndrome). Men were recruited from the outpatient clinic of the Department of Internal Medicine, Division of Endocrinology and Diabetology, and the outpatient clinic of the Department of Urology, Medical University of Graz, Austria, as well as from male hospital staff and male family members of hospital staff. Men were informed about the trial either by a conversation in the outpatient clinic, by written information posted in the respective outpatient clinics or by a telephone call. All patients were informed that participation in the study is voluntary and that refusal to participate as well as stopping at any time without giving reasons, without any consequences is possible. Written informed consent was obtained before carrying out any study-related procedures from all subjects who participated in the study.

### Intervention

Subjects were allocated to the vitamin D or placebo group according to a computer-generated randomization list using a ratio of 1:1. Study medication was placed into numbered bottles according to this computer-generated randomization list. Randomization procedures were conducted using a web-based software (http://www.randomizer.at/) with GCP compliance as confirmed by the Austrian Agency for Health and Food Safety (AGES).

The treatment group received an oral dose of 20,000 IU vitamin D weekly (equivalent to 2857 IU/day) as 50 oily drops weekly (Oleovit D3-drops; Fresenius Kabi Austria GmbH, Linz) for 12 weeks and the placebo group received 50 oily drops without vitamin D for 12 weeks. Placebo oil contained the same oil as Oleovit D3-drops (without vitamin D content) and was delivered by Fresenius Kabi Austria GmbH, Linz. All investigators who enrolled participants, collected data, and assigned intervention were masked to participant allocation.

To improve and verify compliance, patients were asked to return the study medication bottles (full as well as empty bottles) at study end (visit 3).

### Primary outcome

The primary outcome was the change in serum TT levels after 12 weeks of vitamin D supplementation compared to placebo.

### Secondary outcomes

The secondary outcome was the change in endocrine parameters including free testosterone (FT), free androgen index (FAI), sex hormone-binding globulin (SHBG), follicle-stimulating hormone (FSH), luteinizing hormone (LH) and estradiol after vitamin D supplementation compared to placebo.

Further pre-specified secondary outcomes included changes in metabolic parameters (area under the curve (AUC)glucose, and AUCinsulin, insulin resistance, insulin sensitivity, serum lipids), body composition (fat mass and lean mass) as well as sexual, psychological, and physical symptoms after vitamin D supplementation (assessed at baseline and after 12 weeks).

There was no change in study outcomes after the trial had commenced. Although pre-specified as secondary outcome, sexual, psychological, and physical symptoms were assessed only at baseline, as we were not able to document these data at study end due to organizational problems.

Further, we performed subgroup analyses of primary and secondary endpoints in study participants with serum 25-hydroxyvitamin D levels < 50 nmol/l at baseline (not pre-specified).

### Procedures

Basal blood samples for 25(OH)D, PTH, TT, SHBG, LH, FSH, estradiol, glucose, insulin, lipids, and calcium were collected between 8.00 and 9.00 a.m. after an overnight fast. 25(OH)D and TT measured by immunoassays were used for evaluation of inclusion criteria. Biobanking of remaining blood samples was performed by freezing and storing at -80 °C until analysis. Serum levels of 25(OH)D and TT were additionally measured by well-adjusted isotope-dilution liquid chromatography–tandem mass spectrometry (ID-LC–MS/MS) methods in 2018 [18, 19]. 25(OH)D and TT measured by ID-LC–MS/MS were used for statistical analyses. FT values were calculated from TT (measured by ID-LC–MS/MS), SHBG, and albumin according to Vermeulen [20]. The FAI was calculated as TT (measured by ID-LC–MS/MS) (nmol/l)/SHBG (nmol/l) × 100.

Details on procedures and laboratory measurements have been published previously [17].

### Statistical analyses

Details on sample size calculation have been published previously [17].

Continuous data are presented as median with interquartile range and categorical data are presented as percentages. The distribution of data was analyzed by descriptive statistics and Kolmogorov–Smirnov test. Skewed variables were log transformed and rechecked for normal distribution. Student’s *T* test was used for comparisons of baseline characteristics between the vitamin D and the placebo group. Analyses of primary and secondary outcome variables were performed according to the intention-to-treat principle and inclusion of all participants with baseline and follow-up values. Analysis of covariance with adjustments for baseline values was applied to test for differences in the primary and secondary outcome variables between the treatment and the placebo group at study end. All statistical procedures were performed with SPSS version 23 (SPSS Inc., Chicago, IL, USA). A *p* value < 0.05 was considered statistically significant.

## Results

**Fig. 1:**
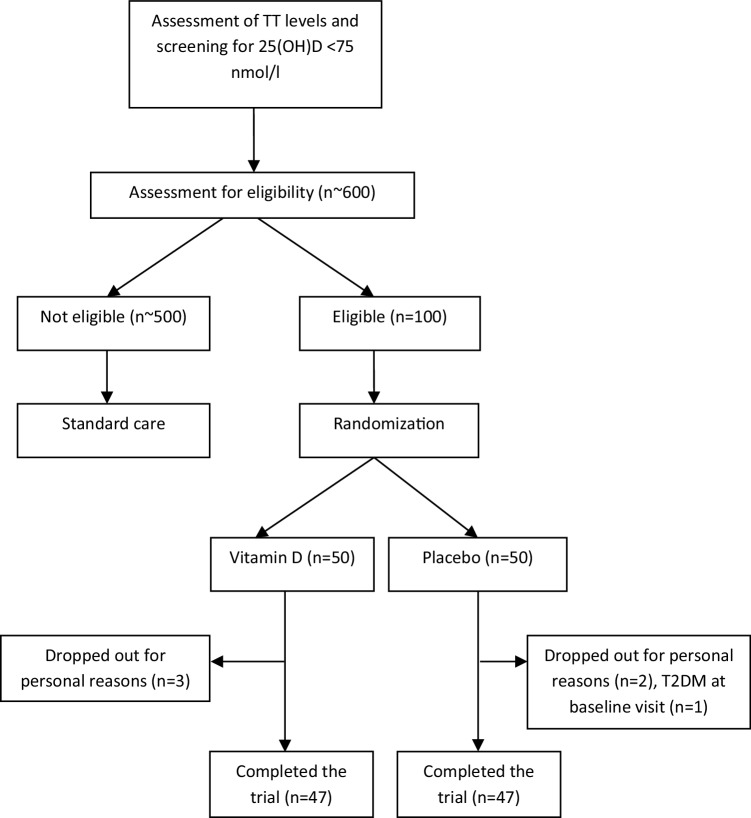
Study flow chart representing recruitment, drop-out, and follow-up of study participants. *TT* total testosterone, *25(OH)D* 25-hydroxyvitamin D, *T2DM* type 2 diabetes mellitus

We took blood samples from approximately 600 men and analyzed 25(OH)D concentrations and TT concentrations (Fig. 1). Men with serum TT levels < 10.4 nmol/l, 25(OH)D levels < 75 nmol/l, and a medical history without any exclusion criteria were informed about the study, its purpose, potential benefits, and possible risks, and were invited to participate in the trial. Main reasons for exclusion were serum TT ≥ 10.4 nmol/l, 25(OH)D levels > 75 nmol/l, as well as refusal to participate. One-hundred men who met all inclusion as well as no exclusion criteria and gave their written informed consent were randomized and enrolled in the study. The first subject was randomized in March 2013 and the last follow-up was performed in November 2017. Baseline characteristics of all study participants are shown in Table 1. We found no significant difference in baseline characteristics between the vitamin D and the placebo group. The mean overall treatment period was 84 ± 4 days in the vitamin D and 85 ± 7 days in the placebo group (*p* = 0.809). A total of 94 men completed the study (Fig. 1) and were analyzed for primary and secondary outcomes.

**Table 1:** Baseline characteristics of study participants

	All study participants (*n* = 100)		Vitamin D (*n* = 50)		Placebo (*n* = 50)		*p* value
	Median	IQR	Median	IQR	Median	IQR	
Age (years)	49	39–56	48	37–56	50	41–58	0.505
BMI (kg/m^2^)	28.6	25.9–32.4	28.4	25.9–31.6	29.4	25.6–34.2	0.534
25-Hydroxyvitamin D (nmol/l)	54	43–69	56	44–72	53	42–63	0.799
PTH (pg/ml)	45.4	35.8–56.4	45.4	35.2–58.0	43.1	36.3–55.2	0.460
Serum calcium (mmol/l)	2.37	2.32–2.43	2.37	2.32–2.44	2.36	2.32–2.42	0.328
Urine calcium (mmol/l)	2.66	1.30–3.63	2.50	1.19–3.61	2.83	1.50–3.63	0.513
Total testosterone^a^ (nmol/l)	12.7	10.6–15.9	12.7	10.5–15.4	13.2	10.7–16.0	0.869
Total testosterone immunoassay (nmol/l)^b^	8.2	6.9–9.3	8.0	6.8–9.1	8.4	7.0–9.6	0.191
Free testosterone (ng/ml)	0.078	0.057–0.094	0.080	0.058–0.092	0.075	0.053–0.09	0.798
SHBG (nmol/l)	28.9	22.9–41.5	27.2	20.3–41.5	30.4	24.0–41.5	0.559
Free Androgen Index	44.3	30.2–58.9	44.4	32.5–56.7	40.8	28.8–61.9	0.859
Estradiol (ng/ml)	34.7	26.9–43.0	34.7	26.9–44.2	34.7	26.2–42.8	0.765
FSH (mU/ml)	5.0	3.4–7.6	5.0	3.9–6.8	5.5	3.3–7.9	0.072
LH (mU/ml)	3.7	2.7–5.5	3.7	2.6–5.0	3.8	2.9–5.7	0.259
HOMA-IR	3.0	2.0–4.8	3.0	1.9–4.4	3.1	2.1–5.4	0.387
HOMA-β	165.3	114.7–237.3	160.1	113.8–229.9	184.6	115.5–241.4	0.138
MATSUDA-index	3.7	2.2–6.2	3.5	2.2–5.3	3.9	2.0–7.4	0.242
QUICKI	0.32	0.30–0.34	0.32	0.31–0.35	0.32	0.30–0.34	0.381
AUCglucose	258.5	223.0–299.3	261.5	233.8–304.5	251.0	217.8–299.3	0.310
AUCinsulin	129	74.6–243.5	126.8	84.9–232.2	136.0	66.8–253.6	0.282
Total cholesterol (mg/dl)	211	178–237	204	164–236	216	192–237	0.212
HDL-C (mg/dl)	50	41–60	50	40–60	51	41–61	0.396
LDL-C (mg/dl)	127	101–153	125	98–146	128	112–157	0.450
Triglycerides (mg/dl)	107	76–162	115	75–152	107	77–177	0.212
Fat mass (kg)	28.0	23.1–36.6	26.2	23.0–34.7	31.1	23.6–37.3	0.700
Lean mass (kg)	58.5	55.1–65.4	57.8	53.5–65.4	58.9	56.8–65.3	0.367
AMS score	28	22–37	29	22–38	28	24–34	0.921
IIEF-EF score	28	22–30	28	21–29	28	23–30	0.339

Comparisons of baseline characteristics between men in the vitamin D and the placebo group were performed using student’s *T* test

*HOMA-IR* homeostasis model assessment-insulin resistance, *QUICKI* quantitative insulin sensitivity check index, *AUC* area under the curve, *HDL-C* high-density lipoprotein-cholesterol, *LDL-C* low-density lipoprotein-cholesterol, *AMS* Aging Male’s Symptoms Questionnaire, *IIEF-EF* International Index of Erectile Function—Erectile Function Questionnaire

^a^Total testosterone measured by ID-LC–MS/MS

^b^Total testosterone measured by immunoassay

### Primary and secondary outcome variables

We show results of analyses of primary and secondary outcomes at study end in Table 2. We found no significant treatment effect on primary and secondary outcome variables.

**Table 2:** Continuous primary and secondary outcome variables at baseline and final follow-up at study end (12 weeks) in study participants with available values at both study visits

	Baseline visit		Study end		Treatment effect		*p* value
	Median	IQR	Median	IQR	Between-group differences with 95% CI		
*Endocrine characteristics*							
Total testosterone (nmol/l)							
Vitamin D (*n* = 47)	12.7	10.5–15.4	12.8	10.2–15.5	− 0.188	− 1.50 to 1.12	0.776
Placebo (*n* = 47)	13.4	10.7–16.1	14.2	10.4–16.4			
Free testosterone (ng/ml)							
Vitamin D (*n* = 46)	0.081	0.058–0.095	0.083	0.059–0.096	0.01	− 0.07 to 0.09	0.827
Placebo (*n* = 46)	0.074	0.053–0.095	0.081	0.057–0.093			
SHBG (nmol/l)							
Vitamin D (*n* = 46)	27.2	20.3–38.3	28.3	19.2–38.1	− 1.7	− 4.2 to 0.9	0.197
Placebo (*n* = 47)	31.2	24.4–41.6	36.0	24.7–45.3			
Free Androgen Index							
Vitamin D (*n* = 46)	45.7	32.5–58.6	46.7	35.0–56.4	2.57	− 3.11 to 8.25	0.371
Placebo (*n* = 47)	37.2	28.5–55.4	38.4	28.1–53.1			
Estradiol (ng/ml)							
Vitamin D (*n* = 47)	35.0	26.9–44.2	36.0	29.0–44.8	− 1.1	− 6.1 to 3.8	0.651
Placebo (*n* = 46)	34.5	24.4–42.8	34.8	29.3–46.3			
FSH (mU/ml)							
Vitamin D (*n* = 47)	5.0	3.9–6.8	5.0	4.1–7.4	− 0.14	− 1.1 to 0.77	0.755
Placebo (*n* = 46)	5.5	3.3–8.2	4.3	3.2–6.4			
LH (mU/ml)							
Vitamin D (*n* = 47)	3.7	2.6–4.9	4.8	2.6–6.0	0.44	− 0.48 to 1.36	0.345
Placebo (*n* = 46)	3.7	2.8–5.7	4.3	3.2–6.4			
*Metabolic characteristics*							
Homeostatic model assessment—insulin resistance							
Vitamin D (*n* = 45)	3.0	1.9–4.4	2.6	1.9–3.9	− 0.3	− 1.0 to 0.9	0.955
Placebo (*n* = 44)	2.9	2.0–5.4	3.0	1.7–5.1			
Homeostatic model assessment-β							
Vitamin D (*n* = 45)	160.1	108.3–229.9	161.3	107.4–215.4	− 0.6	− 54.0 to 52.7	0.982
Placebo (*n* = 44)	178.7	120.5–239.4	157.9	94.3–239.4			
MATSUDA-index							
Vitamin D (*n* = 45)	3.5	2.2–5.4	3.9	2.4–6.7	0.3	− 1.1 to 1.7	0.647
Placebo (*n* = 44)	4.1	1.9–7.5	3.6	2.2–6.5			
Quantitative insulin sensitivity check index							
Vitamin D (*n* = 45)	0.32	0.31–0.35	0.33	0.31–0.35	− 0.01	− 0.03 to 0.01	0.365
Placebo (*n* = 44)	0.33	0.30–0.34	0.32	0.30–0.35			
Area under the curve glucose							
Vitamin D (*n* = 45)	263.0	233.8–305.3	245.9	217.3–285.3	− 8.4	− 28.5 to 11.6	0.404
Placebo (*n* = 44)	252.3	219.9–293.4	264.8	223.8–297.0			
Area under the curve insulin							
Vitamin D (*n* = 45)	128.2	89.4–228.3	131.6	75.6–207.2	− 12.7	− 69.6 to 44.1	0.657
Placebo (*n* = 44)	138.6	65.3–244.4	145.1	78.5–220.9			
*Lipids*							
Total cholesterol (mg/dl)							
Vitamin D (*n* = 44)	207	165–237	192	166–228	− 0.28	− 11 to 11	0.960
Placebo (*n* = 44)	216	194–237	211	175–238			
High-density lipoprotein-cholesterol (mg/dl)							
Vitamin D (*n* = 44)	49	41–60	48	40–62	− 0.88	− 5 to 3	0.650
Placebo (*n* = 44)	51	41–61	53	43–65			
Low-density lipoprotein-cholesterol (mg/dl)							
Vitamin D (*n* = 43)	126	98–146	118	93–149	− 0.32	− 10 to 9	0.948
Placebo (*n* = 42)	127	106–157	129	98–149			
Triglycerides (mg/dl)							
Vitamin D (*n* = 44)	122	78–157	113	78–163	10.7	− 10 to 40	0.480
Placebo (*n* = 44)	107	78–175	115	74–160			
*Body composition*							
Fat mass (kg)							
Vitamin D (*n* = 48)	26.2	23.2–34.7	26.7	22.9–35.1	0.3	− 0.7 to 1.3	0.534
Placebo (*n* = 49)	30.7	22.5–37.0	28.9	23.5–36.1			
Lean mass (kg)							
Vitamin D (*n* = 39)	58.0	54.1–65.4	57.9	53.7–65.2	− 0.2	− 0.8 to 0.3	0.389
Placebo (*n* = 37)	58.7	56.5–65.3	59.1	56.8–63.8			

Treatment effects with 95% confidence interval and *p* values were calculated by ANCOVA for group differences at follow-up with adjustment for baseline values. Data are shown as medians and interquartile range

### Subgroup analyses

Baseline characteristics of study participants with baseline 25(OH)D concentrations < 50 nmol/l (*n* = 39) are shown in Table 3. We found no significant difference in baseline characteristics between the vitamin D and the placebo group in this subgroup. We observed a significant increase in SHBG levels after 12 weeks in the placebo group, whereas SHBG levels remained unchanged in the vitamin D group (Table 4). We observed no significant treatment effect on serum TT and the remaining secondary outcome variables (Table 4).

**Table 3:** Baseline characteristics of study participants with serum 25-hydroxyvitamin D levels < 50 nmol/l at baseline

	All study participants (*n* = 39)		Vitamin D (*n* = 19)		Placebo (*n* = 20)		*p* value
	Median	IQR	Median	IQR	Median	IQR	
Age (years)	47	32–53	46	35–52	49	32–54	0.858
BMI (kg/m^2^)	29.8	26.5–34.2	31.2	26.5–33.9	29.4	26.4–34.3	0.609
25-Hydroxyvitamin D (nmol/l)	41	35–45	41	34–45	41	37–44	0.904
PTH (pg/ml)	49.4	42.2–60.8	49.3	35.7–65.5	51.1	42.4–59.0	0.743
Serum calcium (mmol/l)	2.36	2.31–2.39	2.37	2.34–2.44	2.34	2.30–2.39	0.060
Urine calcium (mmol/l)	3.06	1.96–4.32	2.57	1.19–3.88	3.19	2.69–4.44	0.272
Total testosterone^a^ (nmol/l)	12.2	9.7–16.2	12.0	10.6–16.5	12.3	9.6–16.1	0.910
Total testosterone immunoassay (nmol/l)^b^	8.7	7.0–10.0	8.7	7.3–10.0	8.8	7.0–9.9	0.687
Free testosterone (ng/ml)	0.073	0.056–0.091	0.071	0.058–0.088	0.076	0.053–0.093	0.911
SHBG (nmol/l)	27.4	19.5–42.4	27.1	16.2–44.4	28.0	20.1–42.0	0.559
Free androgen index	39.1	29.0–65.1	39.1	29.3–63.6	37.9	28.8–66.0	0.789
Estradiol (ng/ml)	34.5	26.4–47.4	34.2	26.9–53.4	35.4	24.4–41.7	0.239
FSH (mU/ml)	4.8	3.1–6.8	5.0	3.3–6.8	3.5	2.7–7.7	0.351
LH (mU/ml)	3.4	2.6–4.6	3.4	2.6–4.6	3.3	2.3–5.6	0.633
HOMA-IR	3.1	2.1–5.4	3.1	1.9–5.9	3.2	2.2–5.3	0.948
HOMA-β	179.5	118.7–251.6	131.4	85.0–251.6	196.3	137.3–255.0	0.096
MATSUDA-index	3.9	1.9–5.6	3.6	2.1–5.3	4.1	1.9–5.6	0.689
QUICKI	0.32	0.30–0.34	0.32	0.30–0.35	0.32	0.30–0.34	0.415
AUCglucose	261.5	222.0–304.5	261.3	220.5–312.3	262.0	222.0–287.3	0.370
AUCinsulin	126.8	73.9–216.4	125.2	89.4–222.4	136.0	23.5–210.5	0.961
Total cholesterol (mg/dl)	215	179–233	202	166–233	216	196–235	0.420
HDL-C (mg/dl)	49	41–61	48	42–61	50	41–61	0.818
LDL-C (mg/dl)	130	101–145	129	101–144	131	106–161	0.716
Triglycerides (mg/dl)	131	82–179	129	99–174	139	63–181	0.381
Fat mass (kg)	31.6	25.3–39.9	31.6	25.9–39.9	31.3	23.9–39.9	0.797
Lean mass (kg)	58.0	55.2–65.4	61.7	51.4–65.6	58.0	56.4–62.6	0.897
AMS score	27	21–31	23	19–32	28	25–31	0.114
IIEF-EF score	28	23–30	28	24–30	26	23–30	0.671

Comparisons of baseline characteristics between men in the vitamin D and the placebo group were performed using student’s *T* test

*HOMA-IR* homeostatic model assessment-insulin resistance, *QUICKI* quantitative insulin sensitivity check index, *AUC* area under the curve, *HDL-C* high-density lipoprotein-cholesterol, *LDL-C* low-density lipoprotein-cholesterol, *AMS* Aging Male’s Symptoms Questionnaire, *IIEF-EF* International Index of Erectile Function—Erectile Function Questionnaire

^a^Total testosterone measured by ID-LC–MS/MS

^b^Total testosterone measured by immunoassay

**Table 4:** Continuous primary and secondary outcome variables at baseline and final follow-up at study end (12 weeks) in study participants with serum 25-hydroxyvitamin D levels < 50 nmol/l at baseline and with available values at both study visits

	Baseline visit		Study end		Treatment effect		*p* value
	Median	IQR	Median	IQR	Between-group differences with 95% CI		
*Endocrine characteristics*							
Total testosterone (nmol/l)							
Vitamin D (*n* = 19)	12.0	10.6–16.5	12.7	11.7–15.9	− 0.472	− 3.18 to 2.23	0.725
Placebo (*n* = 20)	12.3	9.6–16.1	15.1	10.4–19.6			
Free testosterone (ng/ml)							
Vitamin D (*n* = 17)	0.071	0.058–0.088	0.083	0.067–0.106	0.05	− 0.11 to 0.02	0.545
Placebo (*n* = 18)	0.076	0.053–0.093	0.087	0.063–0.097			
SHBG (nmol/l)							
Vitamin D (*n* = 17)	27.1	16.2–44.4	26.9	15.4–38.1	− 1.7	− 8.8 to − 1.7	0.005
Placebo (*n* = 19)	28.0	20.1–42.0	37.0	21.9–46.7			
Free androgen index							
Vitamin D (*n* = 17)	39.1	29.3–63.6	48.4	41.1–63.0	7.5	− 4.3 to 19.3	0.205
Placebo (*n* = 19)	37.9	28.8–66.0	40.8	28.1–54.5			
Estradiol (ng/ml)							
Vitamin D (*n* = 17)	34.2	26.9–53.4	37.1	27.6–54.8	3.2	− 7.0 to 13.3	0.532
Placebo (*n* = 19)	35.4	24.4–41.7	34.4	28.1–46.1			
FSH (mU/ml)							
Vitamin D (*n* = 17)	5.0	3.3–6.8	4.8	4.2–6.6	0.50	− 1.11 to 2.12	0.531
Placebo (*n* = 19)	3.5	2.7–7.7	4.6	2.5–8.2			
LH (mU/ml)							
Vitamin D (*n* = 17)	3.4	2.6–4.6	4.4	2.3–5.6	1.3	− 0.52 to 3.05	0.160
Placebo (*n* = 19)	3.3	2.3–5.6	3.7	3.1–6.4			
*Metabolic characteristics*							
Homeostatic model assessment—insulin resistance							
Vitamin D (*n* = 16)	3.1	1.9–5.9	2.6	1.4–4.1	0.0	− 2.2 to 2.2	0.999
Placebo (*n* = 17)	3.2	2.2–5.3	3.0	1.1–5.3			
Homeostatic model assessment-β							
Vitamin D (*n* = 16)	131.4	85.0–251.6	158.7	99.9–227.1	9.2	− 83.0 to 101.5	0.893
Placebo (*n* = 17)	196.3	137.3–255.0	146.2	59.0–323.8			
MATSUDA-index							
Vitamin D (*n* = 14)	3.6	2.1–5.3	3.3	2.1–6.7	− 1.0	− 3.6 to 1.7	0.449
Placebo (*n* = 17)	4.1	1.9–5.6	4.3	2.1–8.1			
Quantitative insulin sensitivity check index							
Vitamin D (*n* = 16)	0.32	0.30–0.35	0.33	0.31–0.36	− 0.02	− 0.07 to 0.02	0.258
Placebo (*n* = 17)	0.32	0.30–0.34	0.32	0.30–0.38			
Area under the curve glucose							
Vitamin D (*n* = 17)	261.3	220.5–312.3	269.3	226.0–277.3	− 6.3	− 36.2 to 48.8	0.766
Placebo (*n* = 17)	262.0	222.0–287.3	265.8	239.5–297.0			
Area under the curve insulin							
Vitamin D (*n* = 16)	125.2	89.4–222.4	172.2	95.3–250.7	27.9	− 54.5 to 110.4	0.494
Placebo (*n* = 17)	136.0	23.5–210.5	120.8	78.5–197.3			
*Lipids*							
Total cholesterol (mg/dl)							
Vitamin D (*n* = 16)	202	166–233	190	175–219	− 0.33	− 17 to 16	0.969
Placebo (*n* = 19)	216	196–235	198	174–238			
High-density lipoprotein-cholesterol (mg/dl)							
Vitamin D (*n* = 16)	48	42–61	43	39–53	− 4.1	− 9.9 to 1.7	0.163
Placebo (*n* = 19)	50	41–61	53	40–64			
Low-density lipoprotein-cholesterol (mg/dl)							
Vitamin D (*n* = 15)	129	101–144	119	91–136	− 0.37	− 15 to 14	0.960
Placebo (*n* = 18)	131	106–161	117	96–153			
Triglycerides (mg/dl)							
Vitamin D (*n* = 16)	129	99–174	114	83–150	18.6	− 37 to 74	0.499
Placebo (*n* = 19)	139	63–181	100	64–159			
*Body composition*							
Fat mass (kg)							
Vitamin D (*n* = 17)	31.6	25.9–39.9	31.9	26.7–35.4	0.1	− 1.8 to 2.0	0.911
Placebo (*n* = 19)	31.3	23.9–39.9	28.9	23.6–37.4			
Lean mass (kg)							
Vitamin D (*n* = 17)	61.7	51.4–65.6	60.6	53.6–65.3	0.13	− 0.9 to 1.1	0.786
Placebo (*n* = 19)	58.0	56.4–62.6	57.8	56.8–63.0			

Data are shown as medians and interquartile range. Treatment effects with 95% confidence interval and *p* values were calculated by ANCOVA for group differences at follow-up with adjustment for baseline values

### Mineral metabolism

Parameters of mineral metabolism at study end in all study participants and in study participants with baseline 25(OH)D concentrations < 50 nmol/l are shown in Tables 5 and 6, respectively. We found a significant treatment effect on 25(OH)D levels at study end in both groups. We observed no significant treatment effect on PTH, serum calcium or urine calcium levels in both groups.

**Table 5:** Parameters of mineral metabolism at baseline and study end (12 weeks)

	Baseline visit		Study end		Treatment effect		*p* value
	Median	IQR	Median	IQR	Between-group differences with 95% CI		
25-Hydroxyvitamin D (nmol/l)							
Vitamin D (*n* = 47)	56	44–72	89	83–110	32	23 to 41	< 0.001
Placebo (*n* = 47)	52	42–63	62	52–76			
PTH (pg/ml)							
Vitamin D (*n* = 46)	45.4	35.2–58.0	48.6	37.3–60.2	− 0.9	− 5.7 to 4.0	0.727
Placebo (*n* = 47)	43.1	36.3–55.2	49.9	37.1–58.2			
Serum calcium (mmol/l)							
Vitamin D (*n* = 47)	2.37	2.32–2.44	2.37	2.30–2.44	− 1.7	− 4.2 to 0.9	0.197
Placebo (*n* = 47)	2.36	2.32–2.42	2.36	2.29–2.41			
Urine calcium (mmol/l)							
Vitamin D (*n* = 46)	2.50	1.19–3.61	2.15	0.96–3.94	− 0.25	− 0.98 to 0.48	0.497
Placebo (*n* = 46)	2.83	1.5–3.63	2.54	1.61–3.58			

Data are shown as medians and interquartile range. Treatment effects with 95% confidence interval and *p* values were calculated by ANCOVA for group differences at follow-up with adjustment for baseline values

**Table 6:** Parameters of mineral metabolism at baseline and study end (12 weeks) in study participants with serum 25-hydroxyvitamin D levels < 50 nmol/l at baseline (*n* = 39)

	Baseline visit		Study end		Treatment effect		*p* value
	Median	IQR	Median	IQR	Between-group differences with 95% CI		
25-Hydroxyvitamin D (nmol/l)							
Vitamin D (*n* = 19)	41	34–45	86	79–95	37	27 to 47	< 0.001
Placebo (*n* = 20)	41	37–44	54	42–62			
PTH (pg/ml)							
Vitamin D (*n* = 17)	49.3	35.7–65.5	45.4	30.6–66.1	− 2.7	− 10.0 to 4.6	0.452
Placebo (*n* = 19)	51.1	42.4–59.0	52.0	46.0–58.2			
Serum calcium (mmol/l)							
Vitamin D (*n* = 17)	2.37	2.34–2.44	2.36	2.32–2.38	− 0.1	− 0.1 to 0.1	0.969
Placebo (*n* = 19)	2.34	2.30–2.39	2.33	2.27–2.39			
Urine calcium (mmol/l)							
Vitamin D (*n* = 17)	2.57	1.19–3.88	1.43	1.11–2.45	− 1.26	− 2.60 to 0.07	0.062
Placebo (*n* = 19)	3.19	2.69–4.44	3.36	2.15–5.59			

Data are shown as medians and interquartile range. Treatment effects with 95% confidence interval and *p* values were calculated by ANCOVA for group differences at follow-up with adjustment for baseline values

During the study, we observed no important harms or unintended treatment effects. No study participant treated with vitamin D had developed hypercalcemia at the final study visit.

## Discussion

In this RCT among men with low serum TT concentrations at baseline, we found no significant effect of vitamin D treatment on serum TT levels or secondary end points. When analyses were restricted to men with 25(OH)D levels < 50 nmol/l, we found a significant increase of SHBG levels after 12 weeks in the placebo group, whereas SHBG levels remained unchanged in the vitamin D group. There was no significant effect on serum TT levels or the remaining secondary outcome parameters in this subgroup.

Our results demonstrating no significant effect on TT concentrations are in line with our previous data from men with normal serum TT concentrations at baseline [17]. Correspondingly, a previous post hoc analysis by Heijboer et al. [21] did not find a significant treatment effect. Heijboer et al. [21] investigated vitamin D effects on TT concentrations in three independent studies involving men with heart failure, male nursing home residents as well as male non-Western immigrants in the Netherlands. In addition, Jorde et al. [22] did not find a significant vitamin D effect on TT concentrations in pooled data from three vitamin D RCTs performed in Tromsö with weight reduction, insulin sensitivity, and depression scores as end points. Recently, Zitterman et al. [23] performed a pre-specified secondary analysis of the EVITA (effect of vitamin D on mortality in heart failure) RCT. The authors analyzed the effect of a daily vitamin D supplement of 4000 IU for 3 years (*n* = 71) vs. placebo (*n* = 62) on TT, SHBG, FT, and bioactive T (BAT) in men with 25(OH)D concentrations < 75 nmol/l. At study end, there was no between-group difference regarding androgen levels and SHBG. Consistently, our findings suggest that previous associations between 25(OH)D and testosterone status may have been rather the consequence of confounding and/or reverse causation than of a causal effect of vitamin D on testosterone status.

Interestingly, in men with 25(OH)D levels < 50 nmol/l at baseline, we observed a significant treatment effect on SHBG, the major carrier protein of testosterone, after 12 weeks of vitamin D supplementation. In detail, SHBG levels remained similar in the vitamin D group, whereas SHBG levels increased in the placebo group. Our findings are difficult to interpret and we cannot exclude that they are caused by chance. As the change in SHBG levels was not accompanied by a change in serum TT or FT levels, the clinical relevance of our finding remains to be determined. Data from men with normal serum TT levels participating the Graz Vitamin D&TT-RCT suggest a significant decrease of SHBG levels after 3 months in the vitamin D group but not in the placebo group [17]. It should, however, be emphasized that no significant treatment effect was found in eugonadal men [17] and when analyzing an RCT it is the comparison between the two treatment arms, that is of interest. Correspondingly, Zittermann et al. [23] observed no treatment effect on SHBG levels in the EVITA trial. Those different results might be explained by different study duration (3 months vs. 3 years), study participants (healthy men vs. men with advanced heart failure) and vitamin D dosing regimens (20,000 IU/week vs. 4000 IU/day) used in the studies. In this context, it should also be noted that previous observational studies found an inverse association of 25(OH)D and SHBG levels [16, 24].

Our results regarding varying serum TT levels assessed via immunoassay and ID-LC–MS/MS supports previous statements on the unreliable results of the currently used immunoassays [25] as well as the need for at least two measurements of low TT levels to confirm the diagnosis of hypogonadism [26]. Due to feasibility reasons, we used TT levels measured once by immunoassay to get immediate results, as MS was not available for these measurements at our department. Nevertheless, in light of the large discrepancies between baseline serum TT levels assessed by different methods (12.7 nmol/l vs. 8.2 nmol/l for ID-LC–MS/MS and immunoassay, respectively), the use of ID-LC–MS/MS for measuring TT levels should be considered for evaluation of inclusion criteria in future studies.

Our results in men with normal serum TT levels suggest an adverse effect of vitamin D supplementation on insulin sensitivity [17]. In contrast, in men with low serum TT levels at baseline we found no significant effect on metabolic parameters including insulin sensitivity. Previous evidence on vitamin D and insulin sensitivity is inconsistent. Observational studies point towards a positive association of vitamin D and insulin sensitivity [2]. Evidence from previous RCTs revealed, however, conflicting results [27, 28]. A positive vitamin D effect has been demonstrated in insulin-resistant, vitamin D-deficient women [28]. Mousa et al. [27] failed to demonstrate a significant vitamin D effect on insulin sensitivity (determined via hyperinsulinemic/euglycemic clamp) in vitamin D-deficient overweight or obese adults, which is in line with our results. We, therefore, cannot exclude that our previous findings [17] were caused by chance.

Our study has several limitations that should be noted. First, we used TT measured by immunoassay for evaluation of inclusion criteria. As TT levels measured by ID-LC–MS/MS were higher than expected, we cannot exclude significant vitamin D effects on androgen levels in men with lower TT levels at baseline. Further, as we investigated a cohort of men with relatively high baseline 25(OH)D levels, we cannot exclude vitamin D effects in men with severe vitamin D deficiency. This notion is supported by the fact that subgroup analyses of men with 25(OH)D levels < 50 nmol/l revealed a significant effect on SHBG levels at study end. Given that a U-shaped association of vitamin D levels with hypogonadism has been observed previously, one might speculate that a RCT aiming at target 25(OH)D levels between 75 and 100 nmol/l would provide different results. Further, time interval of vitamin D supplementation (daily instead of weekly doses) as well as the relatively short treatment period might have had an impact on our study outcome. We cannot exclude substantial effects of vitamin D on androgen levels with different doses, time intervals or longer treatment. As we present results from a single-center study performed in healthy middle-aged men with low serum TT levels, our results may not be generalizable to other populations.

Strengths of our study include the study design of an RCT as well as the use of state-of-the-art and standardized methods to measure 25(OH)D as well as TT concentrations in our samples [26]. Further, we included a relatively large number of participants and the dropout rate was low.

In summary, we found no significant vitamin D effect on androgen levels including TT, FT and FAI concentrations in this cohort of middle-aged healthy men with low baseline serum TT levels. This finding confirms our previous results in men with normal serum TT levels and suggests that vitamin D treatment has no clinical relevant effect on testosterone levels in men. Of note, future studies should only be performed in truly vitamin D-deficient subjects (< 25 or 30 nmol/l) and low testosterone levels to evaluate vitamin D effects on testosterone levels.
